# Relationship between subjective assessment of oral health and medical expenses in community-dwelling elderly persons

**DOI:** 10.1111/j.1741-2358.2011.00459.x

**Published:** 2012-06

**Authors:** Eriko Harada, Shingo Moriya, Ayumi Murata, Masumi Muramatsu, Haruhiko Kashiwazaki, Kunihiko Kobayashi, Kenji Notani, Nobuo Inoue

**Affiliations:** 1 Division of Oral Health Science, Department of Geriatric Dentistry, Graduate School of Dental Medicine, Hokkaido UniversitySapporo, Japan; 2 Department of Oral Health, National Institute of Public HealthWako, Japan; 3 Department of Nursing, School of Nursing, Sapporo City UniversitySapporo, Japan

**Keywords:** oral health, medical expenses

## Abstract

**Objectives:**

The increasing medical expenses of elderly persons in Japan’s rapidly ageing society have become a major concern. It is therefore important to elucidate the factors associated with such escalation. Here, we focused on the relationship between subjective self-assessment of oral health, as an index of general health, and medical expenses (excluding dental repair) under the hypothesis that oral health contributes to general medical expenses. Several studies have shown that oral health status is correlated with general health status among elderly persons. We speculated that oral health status might show a relation with medical costs among elderly persons. However, few studies have investigated this relationship to date.

**Materials and Methods:**

Participants were 259 elderly subjects (range: 65–84 years; 120 men, 139 women) residing independently. Subjective assessment of oral health was evaluated by their responses (‘Good’, ‘Not good’ and ‘Not at all good’) on a survey questionnaire. The correlation between subjective assessment of oral health and medical expenditure was analysed using Spearman’s rank method, the Mann–Whitney *U*-test and the Kruskal–Wallis test. Medical expenses were used as the dependent variable in multinomial logistic regression analysis with background and intraoral factors as independent variables.

**Results:**

A slight yet statistically significant correlation was observed between subjective assessment of oral health and outpatient treatment fees.

**Conclusion:**

The findings revealed that subjective assessment of oral health is significantly and independently related to the medical expenses of community-dwelling elderly persons after adjusting for social background, living environment and physical factors.

## Introduction

As of 2008, the life expectancy of women and men in Japan was 86.05 years and 79.29 years, respectively^1^, and the percentage of people aged ≥65 years reached 24.7%^2^. If the aged population continues to increase, the number of people with chronic diseases will grow, suggesting that health care costs will also escalate. Japan spent 34.1 trillion yen on medical costs in fiscal year 2007, and medical costs rose 3.0% (1 trillion yen) above the level of the previous year^3^. In Japan, most people are covered by a universal health insurance system, and increasing medical costs are becoming a serious social problem. Elucidation of the factors associated with escalating health care costs is thus important.

Several studies have shown that the status of oral health is significantly related to the status of general health (nutritional status, physical performance, functioning in activities of daily living and mortality) among elderly persons^4^–^7^. Therefore, we speculated that oral health status might show a relation with medical costs among elderly persons. However, to the best of our knowledge, only a few studies have investigated the relationship between oral health and medical expenses excluding dental expenses^8^,^9^. Self-assessment of oral health, although subjective, is considered a reliable indicator of oral health status^10^,^11^, and here we attempted to elucidate the relationship between subjective self-assessment of oral health and medical expenses among community-dwelling elderly persons under the hypothesis that oral health contributes to general medical expenses in addition to general health problems.

## Materials and methods

Ethical approval was obtained from the Ethical Committee of Hokkaido University Graduate School of Dental Medicine (Hokkaido, Japan). Written informed consent was provided by each study participant.

### Characteristics of the research area and study population

Tomamae is a town located in the north-west district of Hokkaido, Japan. Its main industries are deep-sea fishing, agriculture and dairy farming. Over a 10-day period in July 2004, from among the residents of Tomamae, 334 persons aged ≥65 years and residing independently received a dental health examination sponsored by Tomamae public authority. All subjects were able to attend the oral assessment by themselves. Of these 334 participants, the present study enrolled 259 persons (77.5%), all of whom were members of the National Health Insurance Program.

### Survey content

1. Questionnaire items concerning patient background were age, sex, current employment (working or not working), type of household (living alone or with family members), educational background (duration <10 years or ≥10 years) and social interaction (yes or no) including participation in social service groups, sports and other hobbies.
2. Further information was also obtained on chronic systemic diseases (presence or absence of one or more chronic complaints including hypertension, cardiac complaints, diabetes mellitus, cerebrovascular complaints, respiratory tract complaints, renal complaints, articular rheumatism and hepatic diseases). Participants were then classified as having severe disease (heart disease or ≥3 of the complaints stated above), moderate disease (two items or one item excluding hypertension from among the complaints stated above) or mild disease (only hypertension or no complaints).
3. Subjective assessment of oral health was evaluated using the following question: ‘What do you think your oral health status is?’ The response options were ‘Good’, ‘Not good’ and ‘Not at all good’.

### Oral examination

The intraoral examination was carried out by four dentists at the Graduate School of Dental Medicine, Hokkaido University. Calibration was conducted to ensure close agreement in the assessment of dental and periodontal status.

1. Periodontal status was evaluated using the WHO Community Periodontal Index of Treatment Needs (CPI) and was examined for all participants with at least one recordable sextant. Each participant was classified according to the maximum CPI code into one of the three groups: codes 0–2, code 3 and code 4.
2. The pattern of occluding pairs of natural teeth (Eichner index) was classified into one of the three classes based on the presence or absence of occluding pairs of natural teeth in the four support zones of the bilateral premolar and molar regions. That is, tooth contact in all four support zones (A), tooth contact in one, two or three zones (B1–B3), or in the frontal region only and absence of tooth contact (B4–C).

### Medical expenses

Medical expenditure values were obtained from National Health Insurance receipts. The total annual cost for each person from August 2004 to July 2005 was calculated as outpatient treatment fees (excluding dental fees) and hospital fees.

### Statistical analyses

The correlations between age, number of natural teeth and medical expenditure were analysed using Spearman’s rank method. The relationships between sex, current employment status, educational background, social interaction, CPI code and medical expenditure were analysed using the Mann–Whitney *U*-test. The relationships between systemic disease, Eichner index, subjective assessment of oral health and medical expenditure were analysed using the Kruskal–Wallis test. Nonparametric tests such as the Mann–Whitney *U*-test and the Kruskal–Wallis test were employed because here medical expenditure did not exhibit a normal distribution.

Outpatient treatment fees were classified into three groups: low (¥0–¥199 999), medium (¥200 000–¥399 999) and high (≥¥400 000). Fees were used as the dependent variable in multinomial logistic regression analysis in which background factors and intraoral factors were employed as independent variables. These models presented the odds ratio (OR) and 95% confidence intervals (95% CI) for the medium and high outpatient treatment fees groups compared with the low group. *p* < 0.05 was considered statistically significant. Statistical analysis was carried out using the spss statistical package (11.0 J for Windows SPSS, Japan, Tokyo).

## Results

Participant background and intraoral factors are shown in Table 1. The correlations between age, number of natural teeth and medical expenditure are shown in Table 2. No significant correlation was observed between age, number of natural teeth and both outpatient treatment and hospitalisation fees. Comparisons of mean outpatient treatment fees according to background factors and oral condition are shown in Table 3. There were significant differences in treatment fees according to sex, current employment status, systemic disease and subjective assessment of oral health. There was no significant difference in hospitalisation fees according to background factors and oral condition.

**Table 1:** Background factors and oral status of the study population

Background factors and oral status	Percentage
Sex	
Female	53.7
Male	46.3
Age (years)	
65–69	22.4
70–74	34.0
75–79	29.7
80–84	13.9
Current employment	
Working	36.3
Not working	63.7
Educational background (duration of study, years)	
≥10	12.7
<10	87.3
Social interaction	
Yes	75.3
No	24.7
Systemic disease	
Mild	37.8
Moderate	32.4
Severe	37.8
Eichner index	
A	12.4
B1–B3	16.2
B4, C	71.0
Number of teeth	
≥14	29.7
13–2	37.5
<1	32.4
CPI code	
3	69.3
4	30.7
Subjective assessment of oral health	
Good	58.7
Not good	32.0
Not at all good	9.3

**Table 2:** Spearman’s rank correlation between age, number of natural teeth and medical expenses

	Outpatient treatment fees		Hospitalisation fees	
		
Background factors and oral status	r	p-value	r	p-value
Age	0.091	0.145	0.049	0.429
Number of natural teeth	−0.028	0.652	−0.047	0.457

r, Spearman rank correlation coefficient.

**Table 3:** Comparisons of mean outpatient treatment fees and hospitalisation fees according to background factors and oral condition

	Outpatients treatment fees (yen)		Hospitalisation fees (yen)	
		
	Mean ± SD (×10^5^)	p-value	Mean ± SD (×10^5^)	p-value
Sex^a^				
Female	2.97 ± 2.22	0.044	1.76 ± 6.76	0.498
Male	2.77 ± 3.02		2.02 ± 7.30	
Current employment status^a^				
Working	2.41 ± 2.25	0.014	1.09 ± 3.45	0.195
Not working	3.15 ± 2.78		2.33 ± 8.36	
Educational background (duration of study, years)^a^				
≥10	2.36 ± 1.55	0.575	3.70 ± 12.37	0.562
<10	2.96 ± 2.73		1.62 ± 5.82	
Social interaction^a^				
Yes	2.82 ± 2.63	0.400	1.81 ± 6.95	0.499
No	3.07 ± 2.60		2.09 ± 7.22	
Systemic disease^b^				
Mild	1.93 ± 2.57	0.000	2.02 ± 7.45	0.229
Moderate	2.92 ± 1.90		1.05 ± 3.25	
Severe	4.04 ± 2.89		2.61 ± 9.10	
Eichner index^b^				
A	1.95 ± 1.46	0.054	1.15 ± 3.71	0.905
B1–B3	3.69 ± 3.39		2.24 ± 10.59	
B4, C	2.86 ± 2.53		1.94 ± 6.45	
CPI code^a^				
3	3.12 ± 2.78	0.169	1.68 ± 5.91	0.212
4	2.53 ± 2.32		1.91 ± 9.67	
Subjective assessment of oral health^b^				
Good	2.61 ± 2.69	0.006	1.84 ± 6.57	0.967
Not good	3.17 ± 2.52		2.28 ± 8.57	
Not at all good	3.61 ± 2.33		0.76 ± 1.77	

a Mann–Whitney *U*-test.

b Kruskal–Wallis test.

Table 4 presents the results of multinomial logistic regression analysis for outpatient medical fees. Chronic medical conditions and subjective assessment of oral health were significantly related to the level of outpatient treatment fees. The ORs for medium and high outpatient treatment fees compared with low outpatient treatment fees were significantly higher for moderate and severe chronic medical conditions than for mild chronic medical conditions. Comparing subjective assessment of oral health of ‘Not at all good’ with ‘Good’, the OR for medium outpatient treatment fees was 5.266 (95% CI 1.489–18.625; *p* = 0.010) and that for high outpatient treatment fees was 4.502 (95% CI 1.093–18.537; *p* = 0.037).

**Table 4:** Multinomial logistic regression analysis for outpatient medical fees

	Outpatient medical fees					
	
	¥200 000–¥399 999			≥¥*400 000*		
		
	OR	95% CI	p-values	OR	95% CI	p-values
Eichner index						
A	1.00			1.00		
B1–B3	1.34	0.426–4.188	0.619	4.36	0.908–20.923	0.066
B4, C	0.96	0.364–2.525	0.931	2.44	0.580–10.282	0.223
Systemic disease						
Mild	1.00			1.00		
Moderate	3.41	1.655–7.013	0.001**	3.44	1.357–8.717	0.009*
Severe	3.84	1.704–7.013	0.001**	11.69	4.589–29.790	0.000**
Sex						
Female	1.00			1.00		
Male	0.59	0.313–1.126	0.110	0.81	0.386–1.679	0.563
Age						
65–69 years	1.00			1.00		
70–74 years	0.94	0.387–2.261	0.883	1.10	0.386–3.116	0.863
75–79 years	1.04	0.407–2.644	0.940	1.36	0.461–4.030	0.576
≥80 years	1.00	0.300–3.313	0.996	0.76	0.187–3.112	0.705
Current employment status						
Working	1.00			1.00		
Not working	1.65	0.799–3.390	0.176	1.36	0.600–3.099	0.459
Participate in social activities	1.00			1.00		
Do not participate in social activities	1.12	0.530–2.382	0.761	0.94	0.400–2.219	0.891
Educational background						
≥10 years	1.00			1.00		
<10 years	0.48	0.198–1.177	0.109	1.72	0.465–6.343	0.417
Subjective assessment of oral health						
Good	1.00			1.00		
Not good	1.36	0.677–2.738	0.387	1.77	0.808–3.886	0.153
Not good at all	5.27	1.489–18.625	0.01*	4.50	1.093–18.537	0.037*

OR (odds ratio) and 95% CI (confidence interval) for outpatient medical fees of ¥200 000–¥399 999 and ≥¥400 000 were calculated compared with ¥0–¥199 999.

* *P* < 0.05;

** *P* < 0.01.

## Discussion

The present study showed significant relationships between subjective assessment of oral health and annual medical expenses (excluding dentistry) after adjustment for dentition status, demographic factors and chronic medical conditions. Correlations between sex, current employment status and medical expenses were observed, but significant relationships were not established in the multinomial logistic regression analysis.

Participation in the present study was dependent upon the voluntary agreement of participants, which resulted in selection bias with respect to oral health concerns. However, the oral health concerns of the participants were considered identical to those of the general elderly population in Japan because the mean expense for dental care in the present study (¥27 940) was almost the same as that reported for persons aged ≥65 years throughout Japan (¥29 800)^12^. However, causal inference is not possible because this study applied a cross-sectional design.

Self-assessment of general health has important practical value as a health evaluation index for the elderly^13^. It is related more to prediction of mortality rate rather than being an objective criterion of health^14^. Subjective assessment of oral health is also considered a comprehensive index reflective of oral status, having been accepted in several studies^15^,^16^. Further, subjective assessment of oral health also reflects oral function, given our previous findings that there is a relationship with subjective well-being and mastication ability based on self-assessment and that subjective assessment of oral health is related to the status of oral cleaning, temporomandibular disorders and denture fitting in women^17^. Consequently, we focused on subjective assessment of oral health to investigate the relationships between oral health status and medical expenses in elderly subjects.

A large-scale epidemiological survey in Japan conducted by the Dental Association of Hyogo Prefecture showed that the number of teeth was significantly negatively related to annual medical expenditure among elderly persons^4^. However, no significant relationship between the number of teeth and medical expenses was observed in the present study. One reason for this difference may be that the subjects from Hyogo Prefecture were limited to only those patients who required dental care.

It has been hypothesised that subjective assessment of oral health is correlated to the impairment of general health^18^. Furthermore, it has also been reported that the number of times a subject receives medical treatment is influenced by medical needs^19^. Medical need has also been suggested as the primary factor determining outpatient medical expenses^20^. Thus, people who have good subjective assessment of oral health also might have good general health. Accordingly, they are less likely to seek consultation from their family doctor or local hospital, thereby incurring lower medical expenses, compared with subjects with poor assessment of oral and general health.

It has been suggested that subjective assessment of oral health has a significant independent effect on psychological well-being^17^ and life satisfaction^21^, while dental health behaviour has been shown to be associated with lifestyle^22^–^24^. Elderly subjects who have good psychological well-being and a good quality-of-life tend to find it easier to maintain their overall health. Medical expenditure in such elderly subjects will therefore be low.

It has also been reported that food selection is substantially affected by oral status^25^ and that people who consume healthy foods tend to have lower medical expenses^26^. This is because people who have good subjective assessment of oral health also have good oral health status; that is, they have better nutrition^27^, thus giving them stronger resistance to chronic or contagious diseases^28^,^29^. Consequently, they may not suffer from medical disorders so readily, which keeps their medical expenses low.

Having a chronic medical condition is considered a major contributing factor to the medical expenses of elderly persons; the present study supports this hypothesis. The relationship between subjective assessment of oral health and medical expenses was established after adjustment for chronic medical conditions. This suggests that subjective self-assessment of oral health is significantly related to medical expenses through mechanisms other than chronic medical conditions. We previously demonstrated that people who have good self-assessment of masticatory ability also have good physical performance^30^,^31^. Self-assessed masticatory ability has been significantly related to subjective assessment of oral health^17^. For the elderly, maintaining muscular strength and balance is very important for protection against functional disorders, fragility, risk of falling and physical disabilities^32^,^33^. It has also been reported that muscular strength training reduces medical expenses^34^. One could argue that subjective assessment of oral health may therefore be related to the maintenance of physical strength, thus preventing a decline in general health. Consequently, subjective assessment of oral health may be related to a decrease in medical expenses.

## Conclusion

We found that, after adjusting for confounding variables, subjective assessment of oral health was significantly and independently related to the medical expenses of community-dwelling elderly persons. These findings suggest that oral health status is an important parameter when elucidating the factors contributing to increased medical expenses among elderly persons.
